# SETDB1 Overexpression Sets an Intertumoral Transcriptomic Divergence in Non-small Cell Lung Carcinoma

**DOI:** 10.3389/fgene.2020.573515

**Published:** 2020-12-02

**Authors:** Yong-Kook Kang, Byungkuk Min

**Affiliations:** ^1^Development and Differentiation Research Center, Korea Research Institute of Bioscience Biotechnology, Daejeon, South Korea; ^2^Department of Functional Genomics, Korea University of Science and Technology, Daejeon, South Korea

**Keywords:** lung cancer, SETDB1, intertumor heterogeneity, epithelial-mesenchymal transition, RNA interference

## Abstract

An increasing volume of evidence suggests that SETDB1 plays a role in the tumorigenesis of various cancers, classifying SETDB1 as an oncoprotein. However, owing to its numerous protein partners and their global-scale effects, the molecular mechanism underlying SETDB1-involved oncogenesis remains ambiguous. In this study, using public transcriptome data of lung adenocarcinoma (ADC) and squamous-cell carcinoma (SCC), we compared tumors with high-level SETDB1 (SH) and those with low-level SETDB1 (comparable with normal samples; SL). The results of principal component analysis revealed a transcriptomic distinction and divergence between the SH and SL samples in both ADCs and SCCs. The results of gene set enrichment analysis indicated that genes involved in the “epithelial–mesenchymal transition,” “innate immune response,” and “autoimmunity” collections were significantly depleted in SH tumors, whereas those involved in “RNA interference” collections were enriched. Chromatin-modifying genes were highly expressed in SH tumors, and the variance in their expression was incomparably high in SCC-SH, which suggested greater heterogeneity within SCC tumors. DNA methyltransferase genes were also overrepresented in SH samples, and most differentially methylated CpGs (SH/SL) were undermethylated in a highly biased manner in ADCs. We identified interesting molecular signatures associated with the possible roles of SETDB1 in lung cancer. We expect these SETDB1-associated molecular signatures to facilitate the development of biologically relevant targeted therapies for particular types of lung cancer.

## Introduction

Lung cancer, which is the leading cause of cancer-related morbidity and mortality worldwide (Jemal et al., 2009), can be categorized into two main clinicopathological categories, namely, small-cell lung carcinoma (SCLC) and non-SCLC (NSCLC). NSCLC accounts for 85% of all lung cancers and is further classified into the following three subtypes: major adenocarcinoma (ADC, ∼40% of all lung cancers), squamous-cell carcinoma (SCC, ∼30% of all lung cancers), and large-cell carcinoma, the occurrence of which is relatively low (Sher et al., 2008). SCC arises from squamous cells in the airway epithelium of the bronchial tubes from the center of the lung, whereas ADC arises from small airway epithelial cells that secrete mucus and other substances (Noguchi et al., 1995). All major histological subtypes of NSCLC are associated with smoking; the occurrence of SCC is strongly associated with smoking compared with ADC, and the latter is the most common histology in never smokers (Sun et al., 2007).

Lung cancer is a molecularly heterogeneous disease, and grasping the underlying biology is essential to develop effective therapies against it. This is especially the case if the relatively higher mutation rates in NSCLC are considered; the mutation frequency has been shown to be 3.5 and 3.9 per megabase (Mb) in ADC and SCC, respectively, and these values are approximately twofold higher than the mean rate of 1.8 per Mb across all tumor types (Kan et al., 2010). A high mutation frequency indicates a broad diversity and heterogeneity within that tumor. The different levels of heterogeneity in cancer are as follows: intertumor heterogeneity, which refers to the diversity between the primary tumor and its metastases, and intratumor heterogeneity, which refers to the subclonal diversity within a single tumor (Burrell et al., 2013; Rich, 2016). Lines of evidence support the fact that lung cancer is composed of subsets of cells and clones with distinct molecular features, even within the same histological subtype (reviewed in Inamura, 2017; Herbst et al., 2018). This tumor heterogeneity, as a source of concern in different tumor responses to treatments, has an impact on the characterization of actionable targets, treatment planning, and drug resistance (Swanton, 2012; Zhang et al., 2014). Therefore, research on tumor heterogeneity should be extended from molecular profiling to epigenetic, phenotypic, and transcriptomic assessment through regional DNA methylation, chromatin state, and RNA and/or protein expression studies over time and during treatment to fully comprehend the impact of tumor heterogeneity on the biology of this cancer type and its impact on the clinical phenotype of patients with cancer (Zhang et al., 2014).

Human cancer genomics studies have recognized that point mutations, translocations, deletions, and gene amplification events frequently occur in genes encoding histone-modifying enzymes such as histone methyltransferases, demethylases, acetyltransferases, and deacetylases (Esteller, 2006; Rodriguez-Paredes and Esteller, 2011). One of the best examples of histone methyltransferases is SETDB1 (SET domain, bifurcated 1). It catalyzes the synthesis of trimethylated histone H3 lysine 9 (H3K9me3), which is a repressive mark to which heterochromatin protein 1 (HP1) is recruited and deposited, thus inducing heterochromatin formation in that region (Schultz et al., 2002). An increasing volume of recent evidence supports that SETDB1 plays a crucial role in the tumorigenesis of various cancers, validating the classification of SETDB1 as an oncoprotein (reviewed in Kang, 2018). SETDB1 gene copy number amplification (CNA) has been frequently observed in various cancers, including melanoma (Ceol et al., 2011), lung SCLC, and NSCLC (Rodriguez-Paredes et al., 2014), hepatocellular carcinoma (Wong et al., 2016), and breast cancer (Regina et al., 2016); a strong correlation between SETDB1 overexpression and cancer development has been detected in various cancers (Kang, 2018). In a study on lung cancer, SETDB1 overexpression correlated with the clonogenicity and tumor size in a xenograft model (Rodriguez-Paredes et al., 2014). In another study (Lafuente-Sanchis et al., 2016), SETDB1 overexpression was proposed to be a prognostic marker to predict tumor recurrence in patients with early stage NSCLC.

Although the findings of studies on SETDB1 have consistently indicated its oncogenic role in various cancers, the broad ranges of genomic targets and protein partners of SETDB1 (usually, transcription factors and associated epidrivers), along with their global effects, make it challenging to prove an immediate connection of SETDB1 with certain tumors. Therefore, the molecular mechanism of SETDB1-involved oncogenesis remains largely unknown. In this study, based on the SETDB1 levels, we attempted subtyping of the lung ADC and SCC samples to measure how SETDB1 overexpression quantitatively and qualitatively alters the global gene expression profiles of ADC and SCC samples. For this, we utilized public lung transcriptome datasets available on The Cancer Genome Atlas (TCGA) site and analyzed the gene sets involved in the cellular processes that are suggested to be associated with SETDB1 function. We identified interesting molecular signatures associated with the roles of SETDB1 in lung cancer and hope that these findings facilitate the development of biologically relevant targeted therapies for particular lung cancer types.

## Materials and Methods

To investigate the effect of SETDB1 overexpression in non-small cell lung cancers (NSCLC), we utilized the genomic datasets of lung adenocarcinoma (LUAD) and lung squamous cell carcinoma (LUSC) deposited in The Cancer Genome Atlas (TCGA, 2018). All plots were generated under R-environment or by MS-EXCEL.

### SETDB1 Gene Copy-Number Alteration and Expression Analysis

To examine the relationship between the *SETDB1* gene copy number and the expression level, the copy-number alteration (CNA) data were download from cBioPortal, and TCGA RNA-seq HTseq-count datasets of LUAD (534 tumor and 59 normal samples) and LUSC carcinoma (503 tumor and 49 normal samples) were downloaded using “TCGAbiolinks” (Colaprico et al., 2016; Mounir et al., 2019). The RNA-seq count data were normalized using DESeq2 (Love et al., 2014), and the *SETDB1* expression levels were extracted. The CNA data and the RNA-seq data were joined together by the sample names, and then the samples were grouped by the types of CNA (amplification, gain, diploid, or loss) and histology (LUAD or LUSC) to generate box plots for the *SETDB1* gene CNA vs. the expression levels. Correlations between SETDB1 CNA and expression in ADC and SCC were calculated using “cor” function with “method = pearson.”

The tumor samples were ranked by *SETDB1* levels and the top 20% (*n* ≅ 100) and the bottom 20% (*n* ≅ 100) of them were arbitrarily chosen as SETDB1-high (SH) and SETDB1-low (SL) group, respectively, for transcriptomic analysis. In ADC samples, the mean expression levels of the selected SH and SL groups were 4,211.15 (ranging from 16,371.35 to 2,983.01) and 1,562.89 (1,723.82–873.64) in normalized counts, respectively, while in SCC samples they were 3,691.52 (13,308.90–3,002.55) and 1,468.37 (1,857.16–1,018.27), respectively. Regarding normal samples, we used all normal samples (*n* = 108 in total) provided in the TCGA ADC and SCC datasets weather they were paired with tumor samples or not. Of 108 samples, 59 were from the TCGA ADC expression datasets and 49 were from the SCC datasets, with 33 and 17 of them paired with the tumor samples.

### Survival Probablility Analysis

For survival analysis, clinical data for TCGA LUAD (ADC) and LUSC (SCC) patients were downloaded using “GDCquery,” “GDCdowload,” and “GDCprepare_clinic” functions in “TCGAbiolinks,” and datasets for SETEDB1 high and low patients were extracted and labeled as “Top100” and “Bot100,” respectively. Survival analysis was performed, and result was plotted using “TCGAanalyze_survival” function in “TCGAbiolinks.”

### Cell Culture, Transfection, and Real-Time PCR

The human NSCLC cell line A549 was maintained in RPMI 1640 medium (Gibco, United States) supplemented with 10% heat-inactivated fetal bovine serum (Hyclone, United States), 2 mM glutamine and antibiotics (100 U/ml penicillin and 100 μg/ml streptomycin) at 37°C in a humidified atmosphere of 5% CO_2_. Cells were transfected with 4 μg of a CMV promoter-driven RFP-SETDB1 expression plasmid per well using Lipofectamine 3000 (Invitrogen) and were observed for RFP signal in fluorescence miscroscope immediately before harvest 48 h after transfection.

Total RNAs were extracted from A549 cells and reverse transcription was performed by incubating 1 μg of DNase I−pretreated RNA with Superscript III enzyme (Invitrogen), 20 μM oligo−dT primers (Invitrogen), and 50 ng random hexamers (Invitrogen) at 50°C for 1 h. Five nanograms of the synthesized cDNAs was used for real−time quantitative PCR (QuantStudio3 Real−Time PCR system, ABI) with the specific primer sets for individual target genes (Supplementary Table S1). PCR was performed with following program: 5 min of pre-denaturation at 95°C followed by 40 cycles of 95°C for 15 s, 55°C for 15 s, and 72°C for 1 min. Finally, relative expression levels of target genes against *GAPDH* gene were calculated using QuantStudio Design and Analysis Software (Thermo).

### Differential Expression Analysis and GSEA

To explore the *SETDB1* expression dependent transcriptomic divergences in lung cancer samples, the TCGA LUAD, or LUSC samples were ordered based on the expression levels of *SETDB1*, and top-ranked and bottom-ranked 100 samples were selected; they were named as SH and SL groups, respectively. For t-distributed stochastic neighbor embedding (*t*-SNE), “Rtsne”^1^, a R wrapper for *t*-SNE, was used with “perplexity = 30” and “pca = true.” Then, transcriptomic data of SH and SL samples were extracted from the earlier DESeq2-normalized expression dataset for the differential expression analysis. For gene set enrichment analysis (GSEA), gene collections for “HALLMARK,” “KEGG pathways,” and “GO Biological Process” from MSigDB v7.0 (Liberzon et al., 2011, 2015) were obtained, and gene sets enriched in either SH or SL group were identified using fGSEA (Korotkevich et al., 2019).

In addition to the fGSEA that was generated based on group means, single sample GSEA (ssGSEA) was performed using gene set variation analysis (GSVA) (Hanzelmann et al., 2013), a R package for gene set variation analysis among individual samples. First, the normalized count data from “DESeq2” were converted to an input matrix for “GSVA” by a home-brew R code, and enrichment scores (ES) for individual samples were calculated by the “gsva” function with “method = gsva.” Then, using “limma” (Ritchie et al., 2015), a R package for differential expression analysis, gene sets with significantly altered activations (FDR < 1 × 10^–5^) were identified, and the results were visualized on volcano plots and heatmaps generated by “plot” and “ggplot” functions in R.

### DNA Methylome Analysis

To compare the methylation states of SETDB1-high and SETDB1-low lung cancer samples, Illumina Human Methylation 450 data (level 3) of TCGA of LUAD and LUSC samples were downloaded using “TCGAbiolinks,” and the pre-processed and scaled methylation beta-values for SH and SL samples were extracted. Next, beta-values were converted to *M*-values for variance stabilization, and differentially methylated CpG sites (DMCs) were identified using “limma” (*p*_*adj*_ < 0.01, log_2_FC > 0.5) and annotated with genomic features (e.g., genes and CGIs, hg38 from UCSC genome browser) using “bedtools” (Quinlan and Hall, 2010). Finally, differential methylation between SH and SL samples was examined by surveying the methylation fold-change distributions of CpGs on annotated CGIs and genes.

## Results

### Enhanced SETDB1 Expression Sets a Transcriptomic Distinction Among Lung Tumors

**FIGURE 1:**
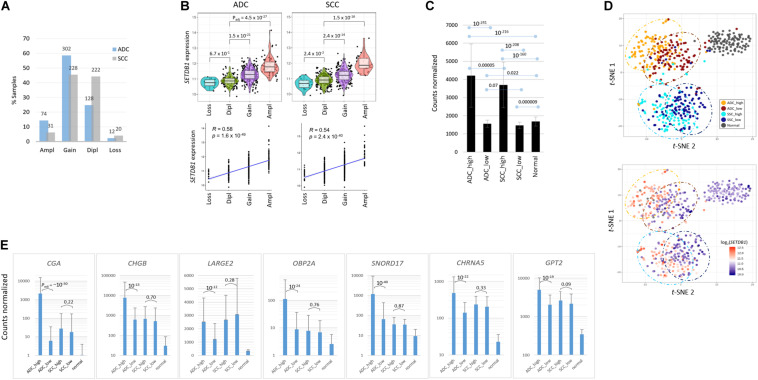
Transcriptomic differences between SETDB1-high and SETDB1-low tumor samples in lung adenocarcinoma (ADC) and squamous-cell carcinoma (SCC). **(A)** The frequency of the *SETDB1* gene copy number alteration (CNA) in The Cancer Genome Atlas (TCGA)-deposited lung ADC and SCC samples. The *X*-axis represents the types of CNAs (Ampl/Gain, more than wild-type copy number; Dipl, wild type; Loss, heterozygous or homozygous deletion), whereas the *Y*-axis indicates the fraction of each CNA type in the lung cancer samples. The actual number of samples is denoted on each bar. Ampl, amplification; Dipl, diploid. **(B)** A high correlation between the *SETDB1* CNA (*x*-axis) and expression (*y*-axis). Violin plots showing *SETDB1* expression levels in samples with *SETDB1* CNA. A statistical significance (Wilcoxon signed-rank test) of each CNA type against the Dipl group is indicated. **(C)** The mean *SETDB1* expression levels in SETDB1-high and SETDB1-low samples of lung cancer groups. Adjusted *p*-values are indicated between sample groups. The error bars indicate standard deviation. ADC/SCC-high/ADC/SCC-low, ADC, or SCC samples with high or low SETDB1 expression levels. **(D)** The *t*-SNE plots of lung cancer samples. Samples are colored by group names (i.e., the *SETDB1* expression level and lung cancer types) in the upper panel and by the SETDB1 expression levels in the lower panel. **(E)** Differentially expressed genes (DEGs) in SETDB1-high ADC tumors. ADC SETDB1-high specific DEGs were chosen between ADC SETDB1-high and normal samples (*P*_*adj*_ < 10^– 50^) for the first round, and the resulting groups of genes were compared between SETDB1-high and SETDB1-low samples in ADCs (*P*_*adj*_ < 10^– 10^) and, simultaneously, in SCCs (> 0.01) for the second round. Adjusted *p*-values are indicated.

In this study, we investigated the effect of increased *SETDB1* transcript levels in the two most frequent histologic subtypes of NSCLC, ADC, and SCC. We inspected the genome and transcriptome data that are publicly available from the cBioPortal and The Cancer Genome Atlas (TCGA). The *SETDB1* gene amplification was more frequent in ADC samples than in SCC ones [14.3% (74/516) vs. 6.2% (31/501); Figure 1A]. We observed a correlation between the *SETDB1* copy number and the *SETDB1* expression level in both types of cancer (*p* < 2.4 × 10^–40^; Figure 1B). We sorted the sample data based on the *SETDB1* levels and, then, classified them into the following two groups: a top-ranked (*SETDB1*-high, SH; *n* = 100) group and a bottom-ranked (*SETDB1*-low, SL; *n* = 100) group. The mean fold difference between the SH and SL groups was 2.69 and 2.53 in the ADCs and SCCs, respectively (Figure 1C). The *SETDB1* level significantly differed between SH group samples and the normal samples, whereas the levels in the SL group samples and the normal samples were close. *t*-Distributed Stochastic Neighbor Embedding (t-SNE) analysis revealed a divergence between the SH and SL samples within each cancer type, along with a vivid distinction between the cancer samples and normal samples or between the ADCs and SCCs (Figure 1D). We examined the effect of SETDB1 overexpression on the survival rates of lung cancer patients and found no significant difference in the 5 years survival rates between SH and SL tumors (Supplementary Figure S1).

To select marker genes that are specific for the SH samples against the SL samples, we first chose genes that are differentially expressed in the ADC-SH and SCC-SH samples compared with the normal samples (*P*_*adj*_ < 1 × 10^–50^), and then, among the chosen genes, we selected the ADC-specific (*P*_*adj*_ < 1 × 10^–10^ for ADCs and > 0.01 for SCCs in the comparison between SH and SL samples) or the SCC-specific SH marker genes (*P*_*adj*_ < 1 × 10^–10^ for SCCs and > 0.01 for ADCs). The ADC markers, including *CGA, SNORD17, SPINK4, OBP2A, BPIFA2, CHGB*, and *SMKR1*, are representatively shown in Figure 1E (see Supplementary Table S2 for the entire gene list).

For validation, we examined another dataset (GSE41271; Sato et al., 2013; *n* = 20 for the SH and SL groups each; fold change = 2.28) from the lung ADC samples publicly available (Supplementary Figure S2A). The dataset was obtained using a microarray platform and thus largely differed in terms of the numbers and types of transcripts from RNA-seq datasets; nevertheless, approximately 40% of the DEGs (21/52; *P*_*adj*_ < 0.00001) were found to be shared with the present dataset (Supplementary Figure S2B). In addition, we transiently transfected A549 LUAD cells with *GFP-SETDB1* expression plasmid, and found from a quantitative real-time PCR that the DEGs that were overexpressed in the SH tumor samples were similarly overrepresented in the GFP-SETDB1 expressing A549 cells compared with the control A549 cells (Supplementary Figure S3). This finding highlights the fidelity of the markers that we detected and suggests that these marker genes serve to discriminate SH tumors from SL tumors, ADCs from SCCs, and lung cancer samples from normal samples in multiple ways.

### Transcriptomic Features of SETDB1-Overexpressing Lung Cancer Samples

**FIGURE 2:**
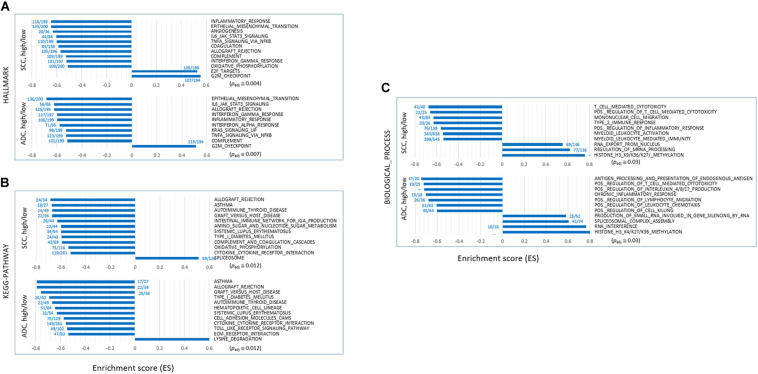
Gene set enrichment analysis (GSEA) of transcriptomes of SETDB1-high samples over SETDB1-low samples. The bar graphs show the enrichment scores (ES) of the GSEA on the “HALLMARK” **(A)**, “KEGG_PATHWAY” **(B)**, and “BIOLOGICAL_PROCESS” **(C)** collections. The ES represents the degree to which the genes in the sets are overrepresented at either the top or bottom of the ranked list of genes. The fractional numbers (blue) indicate the proportion of leading-edge genes relative to the whole genes that belong to the corresponding gene set. The approximate adjusted *p*-values (*P*_*adj*_, Benjamin and Hochberg-corrected enrichment statistic) are indicated.

We performed GSEA on the “HALLMARK,” “KEGG_PATHWAY,” and “BIOLOGICAL_PROCESS” collections using the fast pre-ranked GSEA (fGSEA) package to identify and interpret coordinate changes in the transcriptome of SH samples over the SL samples. When sorted by the enrichment scores [ES; absolute ES value (| ES|) > 0.5] and normalized ES (NES; | NES| > 2.0) that estimate the degree to which the genes in the terms are overrepresented at either the top or the bottom of the ranked list of genes, the selected terms mostly showed negative ES/NES values. It indicated that those genes in the designated collections were mostly underrepresented in the SH samples in both the ADC and SCC groups (Figures 2A–C and see Supplementary Table S3). Overall, the genes in the inflammation- and immune response-related terms including “TNFA_SIGNALING_VIA_NFKB,” “INTERLEUKIN_PRODUCTION,” “INTERFERON_GAMMA_ RESPONSE,” “ALLOGRAFT_REJECTION,” “COMPLEMENT,” and “ANTIGENE_PROCESSING_AND_PRESENTATION” were significantly underrepresented in the SH samples, whereas those in the “G2M_CHECKPOINT,” “DNA_REPLICATION_CHECKPOINT,” and “E2F TARGETS” terms were overrepresented. Some terms are notable because they were previously shown or hypothesized to be a function of SETDB1, which we have summarized below.

#### Depletion of Genes Related to Epithelial–Mesenchymal Transition (EMT)

**FIGURE 3:**
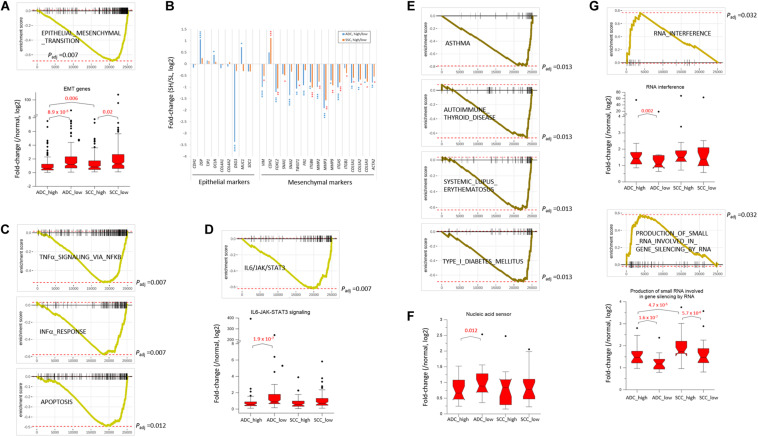
SETDB1-related gene collections from fast pre-ranked gene set enrichment analysis. The GSEA mountain plot shows a significant enrichment **(G)** or depletion **(A,C–E)** of genes for indicated collections in lung SETDB1-high tumors that are presumably related to the SETDB1 function. Light (ADCs) and dark brown lines (SCCs) indicate the running enrichment scores across the fold change-ranked genes in comparison between the RNA-seq gene-level expression of SETDB1-high over SETDB1-low samples. Black vertical tick marks below or above the curve indicate the location of individual target genes within the fold change-ranked gene list. Adjusted *p*-values (*P*_*adj*_, Benjamin and Hochberg-corrected enrichment statistic) are indicated. In **(B)** the fold change (SETDB1-high vs. SETDB1-low tumors) levels of epithelial marker genes and mesenchymal marker genes in the “epithelial–mesenchymal transition” (EMT) gene set in ADCs (blue) and SCCs (red). Asterisk indicates adjusted *p*-values (* < 10^– 2^; ** < 10^– 4^; *** < 10^– 6^; **** < 10^– 10^). The box plots in **(A,D,F,G)** compare the normalized expression levels of the leading-edge genes of the denoted gene sets between SETDB1-high vs. SETDB1-low tumors of ADCs and SCCs (*p*-values, paired sample *t*-test).

NSCLC mainly arises from the bronchial and alveolar epithelium; thus, it is speculated that EMT plays an important role in the biological behavior of NSCLC and serves as a key mechanism in cancer cell metastasis (Welch and Hurst, 2019). The genes appertained to the “EMT” collection were markedly underrepresented in the SH samples of both ADC and SCC groups (Figure 3A). The mean expression levels of the leading-edge genes (the core of a gene set accounting for the enrichment signal) of the “EMT” collection significantly differed between the SH and SL samples, particularly in the ADCs. Although the transcript levels of the epithelial markers in the SH samples relative to those in the SL samples varied largely, all the mesenchymal markers, except *CDH2/NCAD*, were significantly underrepresented in the SH samples in both ADCs and SCCs (Figure 3B). In line with this, the genes in the “IL6_JAK_STAT3_SIGNALING” and “TGF_BETA_SIGNALING” collections that are known to induce cancer cell metastasis (Chang et al., 2013; Bellomo et al., 2016) were also depleted (Supplementary Table S3 and see below) in the SH samples. Our results strongly suggest an inverse relationship between the SETDB1 levels and EMT in lung tumors, which is in disagreement with the notion that overexpressed SETDB1 promotes metastasis, as reported in liver (Fei et al., 2015; Wong et al., 2016) and breast cancers (Weigelt et al., 2010; Ryu et al., 2019); this probably indicates that SETDB1 behaves differently in different cancers.

#### Depletion of Genes Associated With Host Innate Immune Response

The upregulation of *SETDB1* is suggested to be a common mechanism in tumors to avoid the host innate immune response and apoptosis through a tighter surveillance of transposable element (TE) expression (Cuellar et al., 2017; Kang, 2018). This theory harmonizes well with the reduced expressions of genes in the SH samples that belong to the “INTERFERON_ALPHA_RESPONSE,” “TNFα_ SIGNALING_VIA_NFKB,” and “APOPTOSIS” collections (Figure 3C). The genes in the “IL6_JAK_STAT3_SIGNALING” set were underrepresented in the SH samples (Figure 3D), and the fold-change difference in expression level of leading-edge genes was remarkable between the SH and SL samples of ADCs. Because the components of the IL6/JAK/STAT3 pathway are aberrantly hyperactivated in various tumors and targeting these components can inhibit tumor growth (Johnson et al., 2018), lung cancer cells in which the levels of SETDB1 are high are probably supposed to have a natural ability to control the IL6/JAK/STAT3 pathway, creating an environment that is unfavorable for tumor growth.

#### Depletion of Autoimmunity-Related Genes

Genes in the autoimmunity-related collections (“ASTHMA,” “AUTOIMMUNE_THYROID_DISEASE,” “SYSTEMIC_LU PUS_ERYTHEMATOSUS,” “TYPE_I_DIABETES_MELLITUS,” etc.) were largely underrepresented in the SH samples (Figures 2B, 3E). This suppression of autoimmunity is consistent with a recent hypothesis stating that SETDB1 overexpression tightly seals off the expression of various genomic TEs, thus blocking self-retroelement-derived nucleic acids (RdNAs) to be piled up in the cytoplasm, which can cause autoimmune diseases such as Aicardi-Goutieres syndrome (Volkman and Stetson, 2014). Notably, the genes encoding various nucleic acid sensors involving *TBK1, MAVS, IFI16, STING1, MDA5, RIG1, DDX41*, and *TLR1-TLR10* that patrol the cytoplasm and induce type-I interferon responses were significantly underrepresented (*p* = 0.012, paired sample *t*-test) in the SH samples in the ADCs (Figure 3F). The underlying mechanism that links SETDB1 overexpression to the downregulation of nucleic acid sensors in ADCs should be elucidated in the future.

#### Enrichment of RNA Interference-Related Genes

SETDB1 also participates in the RNA interference pathway, particularly in transcriptional gene silencing (TGS) in association with ARGONAUTE-1 and -2 (Cho et al., 2014). The genes involved in the “RNA_INTERFERENCE (RNAi)” and “PRODUCTION_OF_SMALL_RNA_INVOL VED_IN_GENE_SILENCING_BY_RNA” collections were overrepresented in the SH samples (Figure 3G). In line with this, various gene sets related to “small RNA” were enriched (*p* < 0.002) in the ADC-SH samples with positive ES values: “RNA_METABOLIC_PROCESS,” “PRE_MIRNA_PROCESSING,” “PRODUCTION_OF_SMALL_ RNA_INVOLVED_IN_RNA_INTERFERENCE/GENE_SILENC ING_BY_RNA,” “RNA/NCRNA_EXPORT_FROM_NUCLEUS,” etc. (Supplementary Table S3: BIOLOGICAL_PROCESS) We speculate that these collections are partly associated with an increased production of endogenous small interfering RNAs and their busy nucleocytoplasmic trafficking to target loci through RNAi pathway in the SH cancer cells (Kang, 2018) although it is unknown how SETDB1 is implicated in the production of small RNAs.

**FIGURE 4:**
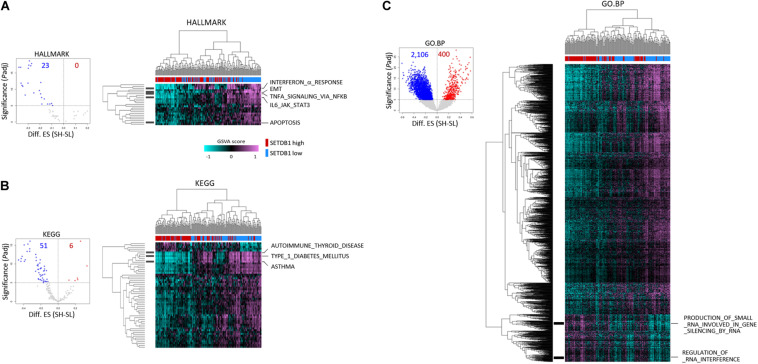
Single sample gene enrichment analysis (GSEA) with gene sets showing differential enrichments in SETDB1-high (SH) and SETDB1-low (SL) lung adenocarcinoma samples. Using GSVA, single sample GSEA was performed on three gene collections from MSigDB (v7.0): HALLMARK **(A)**, KEGG **(B)**, and GO:BP **(C)**. Volcano plot shows the distribution and the number of gene sets with differential enrichments (DE; *FDR* < 1 × 10^– 5^) between the SH and SL samples in each collection; each dot indicates a gene set in selected MSigDB collections. In the plot, *x*-axis designates the difference (SH-SL) of enrichment scores between SH and SL and *y*-axis represents the significance of DE scores; red and blue dots indicate gene sets that are enriched and depleted in SH samples, respectively. Heatmaps show the differential enrichments among individual SH and SL samples. Samples are hierarchically clustered on *x*-axis (SH, red; SL, blue), and significant DE gene sets are shown on *y*-axis. Black bars on the left represent the gene sets mentioned in Figure 3, and the names of the gene sets are denoted on the right. Colors in GSVA score bar indicate enrichment scores in individual samples.

To further test if the ADC and SCC samples could be divided by *SETDB1* level, we performed single sample GSEA using gene set variation analysis (GSVA), which assesses separate enrichment scores for each pairing of a sample and gene set (independent of phenotype labeling) to find out to what extent the genes in a certain gene set are coordinately upregulated or downregulated within a sample (Barbie et al., 2009). The GSVA enrichment scores for the selected gene sets (*FDR* < 1 × 10^–5^) showed that, consistent with the result in Figure 3, the SH samples showed a depletion of genes in most of the selected gene sets in the ADCs (Figure 4) and also in the SCCs (Supplementary Figure S4). We found the gene sets which we have noticed from the bulk GSEA result (Figure 3) being reproducibly depleted or enriched in the GSVA result.

### Expression of the Histone Modification Category Genes in the SETDB1-High Lung Cancer Samples

**FIGURE 5:**
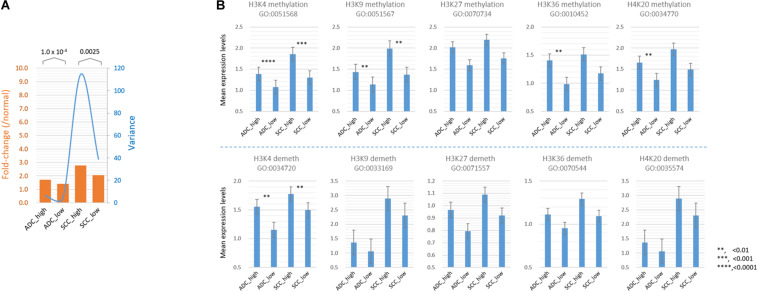
Comparison of the mean expression levels of genes in different histone modification categories between SETDB1-high and SETDB1-low samples in lung ADCs and SCCs. **(A)** The mean expression levels of genes (fold change; orange bars) and variance of the fold change values (blue line) in the histone modification category genes. **(B)** The mean expression levels of “HISTONE METHYLATION” and “HISTONE DEMETHYLATION” category genes. The gene ontology (GO) identification is indicated below each GO term. Asterisks indicate the significant differences at the denoted levels. The expression levels of individual genes were normalized to the mean expression levels of normal samples. A significant difference (paired sample *t*-test) was observed between the SETDB1-high and SETDB1-low samples of ADCs and SCCs.

Because SETDB1 is one of epigenetic players, or epidrivers, that function as a writer, reader, and/or eraser of chromatin marks (Park et al., 2017), the altered expression of SETDB1 may affect other associated epidrivers and their epigenomic signatures. In agreement, the GSEA results revealed that histone lysine (H4K20, H3K27, H3K4, H3K36, and H3K9) methylation-related genes were enriched in the SH samples (Figure 2C). We explored the sub-categories of the “HISTONE_MODIFICATION” collection for altered gene expression, and the result is summarized in Table 1. Collectively, the mean expression levels of the genes in these classes were higher in the SH samples than those in the SL samples in both ADCs and SCCs (Figure 5A). The variance of the expression levels of individual histone modification genes was incomparably high in the SCC-SH samples, which indicates an enormous discrepancy and heterogeneity among the SCC samples.

**TABLE 1:** The mean expression levels of epidriver genes in different “histone modification” categories (GO DAVID).

Histone/DNA modification (GO ID)	No. gene	Mean expression level^*a*^ (SD)				ADC-SH vs. ADC-SL^*b*^	SCC-SH vs. SCC-SL^*b*^	ADC-SH vs. SCC-SH^*b*^	ADC-SL vs. SCC-SL^*b*^
		
		ADC-SH	ADC-SL	SCC-SH	SCC-SL				
Acetylation (GO:0016573)	134	1.711 (3.073)	1.627 (3.554)	5.123 (38.413)	4.505 (31.636)	0.6453	0.3187	0.2985	0.2872
Deacetylation (GO:0016575)	71	1.140 (0.461)	1.084 (0.515)	1.228 (0.651)	1.164 (0.599)	0.1747	0.0310	0.0439	0.0660
Lysine methylation (GO:0034770)	122	1.508 (0.960)	1.170 (0.645)	1.921 (1.685)	1.388 (1.038)	2.8E–10	1.2E–08	0.0002	0.0016
Lysine demethylation (GO:0070076)	27	1.276 (0.847)	1.014 (0.365)	2.079 (3.528)	1.711 (2.801)	0.0188	0.0162	0.2671	0.2010
Arginine methylation (GO:0034969)	8	1.868 (1.203)	1.587 (0.835)	1.828 (1.047)	1.928 (1.205)	0.2734	0.4130	0.9294	0.1483
DNA methylation (GO:0044728)	84	2.723 (4.132)	1.666 (1.439)	7.526 (26.109)	4.229 (14.471)	0.0048	0.0207	0.0893	0.0976
Phosphorylation (GO:0016572)	36	2.132 (2.649)	1.998 (2.318)	3.149 (5.130)	3.140 (5.138)	0.3902	0.9445	0.0317	0.0271
Ubiquitination (GO:0016574)	45	1.487 (1.351)	1.289 (1.181)	1.936 (2.815)	1.592 (1.882)	0.0003	0.0285	0.0591	0.0152
Whole (SD)	527	1.720 (2.485)	1.412 (2.071)	2.763 (10.707)	2.067 (6.236)	0.0001	0.0025	0.0198	0.0084

**^*a*^Normalized by the mean levels of genes of normal samples. ^*b*^p-values (paired sample t-test). SH and SL, SETDB1-high and SETDB1-low, respectively. SD, standard deviation.**

As shown in Table 1, the expression levels of the “HISTONE_LYSINE_METHYLATION” and “HISTONE_LYSINE_DEMETHYLATION” category genes differed (*p* < 0.02) between the SH and SL samples in both ADCs and SCCs, and the “HISTONE_UBIQUITINATION” genes differed in the ADCs (Supplementary Table S4). We further divided the “HISTONE_METHYLATION” category because of its largest size (122 genes) and the most significant difference (*p* < 1.2 × 10^–8^, paired sample *t*-test) between the SH and SL samples. The genes in the H3K4, H3K9, H3K36, and H4K20 methylation categories significantly differed (*p* < 0.01) in the ADCs, whereas only H3K4 and H3K9 category genes differed in the SCCs (Figure 5B). The increase in the expression levels of H3K4 methylation genes was prominent in both the cancer groups, but the upregulation of the H3K4 demethylation category genes was also significant, which could offset the effect of the former on the premise that they share the same sphere of influence over the genome. Conversely, the H3K9 demethylation category genes did not differ between the SH and SL samples, suggesting that both the cancer groups can appreciate the entire impact of the increased expression of the H3K9 methylation genes.

We examined the genes (*n* = 154) encoding effector enzymes and mediators that directly participate in the modification of nucleosomes (Park et al., 2017; Min et al., 2018). Table 2 presents the differentially expressed epidriver genes between the SH and SL samples in the ADCs and SCCs (*p* < 1 × 10^–7^ in either ADCs or SCCs (also see Supplementary Table S5). *HR, SETD5, KMT5B, and KDM6B* genes were differentially expressed only in the ADCs, whereas *CBX1*, *RNF20*, and *RNF38* genes were differentially expressed only in the SCCs; these sets of genes might contribute to the production of characteristic epigenome signatures of ADCs and SCCs, respectively.

**TABLE 2:** The mean expression levels of differentially expressed epidriver genes in either of ADCs or SCCs.

Category	Gene ID	Expression level					Fold-change (SH/SL)*		*P_*adj*_-value* (SH vs. SL)	
				
		ADC-SH	ADC-SL	SCC-SH	SCC-SL	Normal	ADC	SCC	ADC	SCC
K meth	*SETDB1*	4,211.4	1,560.8	3,693.6	1,467.7	1,678.6	1.430	1.331	9.7E-193	1.1E-208
DNA meth	*DNMT3A*	3,566.2	1,881.1	4,013.7	2,410.1	1,407.5	0.922	0.736	4.8E-28	3.0E-17
K meth	*EHMT2*	4,885.8	2,705.0	4,936.3	3,392.1	2,303.2	0.851	0.541	8.1E-25	1.5E-11
K demeth	*KDM5B*	7,773.7	4,439.9	8,035.3	6,011.6	2,906.4	0.808	0.419	1.1E-24	6.1E-08
K meth	*ASH1L*	6,641.9	3,247.3	5,539.5	2,899.1	3,993.7	1.036	0.934	7.5E-24	2.3E-27
K meth	*KMT5B*	3,500.9	2,412.2	2,885.9	2,477.1	2,600.2	0.536	0.220	1.2E-16	3.0E-03
K meth	*KMT2D*	7,272.7	4,065.7	6,393.4	4,556.7	6,288.7	0.839	0.489	2.0E-15	3.2E-06
PRC	*CBX2*	1,295.1	473.2	2,844.2	1,365.9	180.0	1.448	1.058	1.0E-13	2.4E-09
K meth	*SETD6*	911.3	602.8	889.4	662.5	448.7	0.596	0.425	9.7E-13	5.6E-07
DNA meth	*TET3*	2,199.7	1,379.0	4,603.2	3,046.6	1,486.0	0.675	0.595	7.9E-12	2.5E-09
DNA meth	*DNMT3B*	435.6	207.0	827.7	505.3	88.1	1.073	0.712	8.0E-12	1.1E-05
K meth	*SETD5*	6,065.4	4,280.1	5,645.4	4,728.0	4,195.0	0.503	0.256	9.1E-12	4.5E-04
PRC	*ASXL1*	4,446.9	3,265.4	4,486.6	3,621.0	4,054.7	0.443	0.309	4.8E-11	8.9E-05
K meth	*EHMT1*	3,235.4	2,301.4	3,920.8	2,840.5	2,550.2	0.490	0.465	9.1E-11	1.8E-10
K demeth	*HR*	68.6	173.7	3,183.4	2,542.4	163.7	−1.339	0.324	3.5E-10	1.9E-01
K demeth	*PHF8*	2,813.9	1,902.2	3,682.3	2,463.7	1,770.8	0.564	0.580	5.8E-10	1.1E-10
Acetyl	*KAT7*	2,988.2	2,218.8	3,049.2	2,307.9	2,316.7	0.430	0.402	1.9E-09	2.5E-09
K meth	*SETD1B*	2,504.0	1,777.3	2,818.1	2,036.4	2,043.2	0.493	0.469	1.9E-09	2.9E-09
Acetyl	*KAT2A*	4,042.1	2,634.1	4,291.0	3,146.7	1,159.1	0.615	0.447	7.4E-09	3.2E-06
K meth	*KMT2B*	4,319.8	2,877.6	5,682.8	3,593.2	2,547.8	0.586	0.661	1.0E-08	6.5E-12
R meth	*PRMT5-AS1*	25.6	14.1	19.5	18.5	8.5	0.871	0.072	2.0E-08	7.2E-01
K demeth	*KDM6B*	3,318.4	2,259.2	2,954.0	2,887.4	6,097.4	0.554	0.033	8.2E-08	8.5E-01
Acetyl	*KAT6B*	1,835.0	1,336.1	2,149.9	1,494.4	2,369.4	0.460	0.525	2.4E-06	3.9E-09
K meth	*SETD1A*	2,409.6	1,796.4	2,810.9	2,043.8	1,773.9	0.422	0.460	4.7E-06	1.1E-08
K demeth	*KDM3B*	5,409.5	4,359.2	4,703.8	3,374.5	5,018.0	0.309	0.479	8.7E-06	1.0E-14
PRC	*CBX5*	7,212.4	5,238.4	7,017.1	4,677.5	4,799.1	0.462	0.585	9.4E-06	1.2E-09
K demeth	*KDM2B*	1,648.9	1,338.5	2,501.5	1,884.1	1,661.2	0.300	0.409	1.2E-05	3.8E-09
Acetyl	*KANSL1*	3,698.6	2,872.8	3,717.8	2,536.9	2,862.3	0.366	0.551	1.9E-05	2.3E-14
K meth	*EZH1*	1,829.4	1,472.2	1,657.1	1,194.5	2,514.6	0.312	0.472	5.4E-05	1.0E-09
PRC	*CBX1*	4,579.3	3,670.7	6,577.2	4,517.0	2,268.0	0.317	0.542	9.0E–04	1.4E-10
Ubiquit	*RNF20*	2,404.6	2,146.8	3,226.9	2,432.3	2,721.8	0.161	0.408	2.2E-02	4.7E-10
Ubiquit	*RNF38*	2,095.4	1,907.5	2,493.1	1,749.1	3,353.6	0.136	0.511	1.6E-01	4.3E-09

*Log_2_ scale. Meth and demeth, methylation and demethylation; Acetyl and Deacetyl, acetylation and deacetylation; Ubiquit, ubiquitination; R meth, arginine methylation; PRC, Polycomb repressive complex. SH and SL, SETDB1-high and SETDB1-low, respectively.

### Opposite Pattern of DNA Methylation Change in ADC and SCC SETDB1-High Samples

**FIGURE 6:**
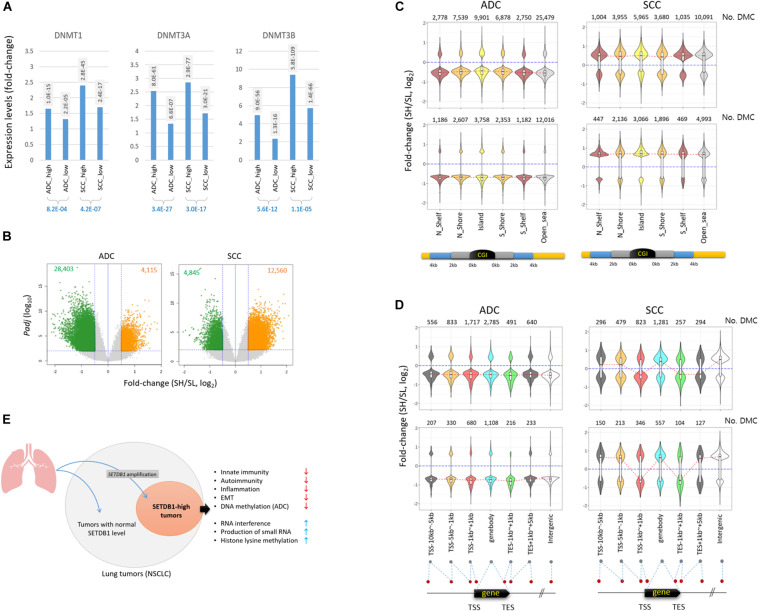
Different pattern of methylation change in ADC and SCC SETDB1-high samples. **(A)** The expression of DNA methyltransferase genes in lung cancer samples. The adjusted *p*-values (*P*_*adj*_) between each cancer group and normal group (shaded) and between the cancer groups (blue) are shown. **(B)** The volcano plots show the differentially methylated CpGs (DMCs) between SETDB1-high (SH) and SETDB1-low (SL) in TCGA LUAD/LUSC methylome data (Infinium HumanMethylation450 BeadChip). The methylome datasets were derived from the same patients’ tumors that we included in the transcriptome analysis in this study. The horizontal blue line indicates the threshold for significance (*P*_*adj*_ = 0.01), whereas the two vertical blue lines represent the fold change cutoff (log_2_FC = 0.5). The number of hypermethylated CpGs in each group is denoted on the top of the plots. **(C)** The distribution of DMCs on CpG islands (CGIs) and surrounding regions in lung cancer samples. The violin plots show the fold change levels of significant DMCs in CGIs, shores, and shelves, as schematically depicted in the plots. **(D)** The distribution of DMCs in genic regions. The violin plots show the fold change levels of significant DMCs on genes and around the transcription start/end site (TSS/TES). The number of DMCs in each region is denoted in each plot. The red dotted lines connect the median fold change values of each region. The blue lines indicate the fold change = 0. **(E)** Illustration of the generation of *SETDB1* level-based, different NSCLC molecular subtype and its singular features in biological processes.

The genes belonging to the DNA methylation category also showed a significant difference between the SH and SL samples (Table 1), with the highest root-mean-square deviation (RMSD) value among the “HISTONE_MODIFICATION” sub-categories (Supplementary Figure S5), which measures how far off the genes in the cancer samples are from those in the normal samples in terms of the mean expression levels (Kwon et al., 2015; Min et al., 2018). We observed that DNA methyltransferase genes such as *DNMT1, DNMT3A*, and *DNMT3B* were overrepresented in all cancer groups, with a significant difference between the SH and SL samples in both ADCs and SCCs (Figure 6A and also see Table 2), which prompted us to explore the genome for the methylation states. We looked into the public 450 K BeadChip array methylome data obtained from the same tumor samples as those used in the present transcriptome study. The volcano plots showed that the methylation levels at individual CpG sites were mostly reduced in the SH samples in the ADCs, whereas they were increased in the SH samples in the SCCs (Figure 6B).

The antithetical pattern of the methylation change was also observed around the CpG island (CGI) regions. In the ADCs, the differentially methylated CpGs identified between the SH and SL samples (DMCs; *P*_*adj*_ < 0.01 and fold change > 1.5) were mostly of lower methylation in the SH samples, whereas they were of higher methylation in the SCCs (Figure 6C). The pattern was uniform because it was observed at all sub-CGI compartments such as shelfs, shores, and islands as well as in the “open_sea” regions. The DMCs in the genic regions involving distal and proximal promoters, gene body, and 3’ untranslated regions (3’-UTRs) were, similar to the CGI DMCs, largely at undermethylated states in the ADC-SH samples (Figure 6D). In contrast to the overall consistent and highly biased methylation change in the ADCs, DNA methylation appeared rather randomly altered in the SCCs, considering the similar fractions of DMCs with increasing and decreasing methylation levels. Based on the pattern of methylation change in the lung cancer samples, we assume that SETDB1 overexpression is associated with the uniform change in genomic methylation (i.e., methylation loss) in the ADCs but not in the SCCs, and the underlying mechanism of this is yet to be elucidated. In conclusion, our findings indicate that *SETDB1* overexpression causes changes in the lung cancer transcriptomes and the methylomes, drawing a line between the SH and SL samples and, as a possible result, leads to a within-a-tumor divergence of cancer cells to a distinctive subpopulation(s), as illustrated in Figure 6E.

## Discussion

This study presented a “within-a-tumor” subtyping result for ADC and SCC samples. The SH tumor samples were distinguished from the SL tumor and normal samples based on the transcriptomes, and these transcriptomes showed various molecular signatures and interaction networks that are possibly related to SETDB1 functions. Because the SL samples did not greatly differ from the normal samples based on the *SETDB1* level alone, the SH sample group might be more diverged than the SL group among the ADC and SCC sample populations (Figure 1D). The SH vs. SL difference was larger in the ADCs than in the SCCs at several points, which are as follows: the fraction of the *SETDB1* gene CNA samples; the mean expression levels of *SETDB1* in the SH samples; the overall ES values and the mean expression levels of leading-edge genes on the GSEA results (Figures 2, 3, respectively); the number of SH vs. SL DEGs (Supplementary Table S2); and finally, the number of DMCs and the pattern of DNA methylation change (Figure 6). Currently, the factors that cause such a difference between the SH samples of ADCs and SCCs are unknown; we assume that ADCs are more sensitive to an altered level of SETDB1, and the histologic difference between the ADCs and SCCs may explain their different sensitivities (Lin et al., 2017). Meanwhile, the SH vs. SL comparison performed in this study is not synonymous with the SH vs. normal comparison because the SL samples, with respect to the transcriptome, were not similar to the normal samples at all and to the SH samples.

The regulation of immunity through the control of TE expression is an emerging research field in cancer, and SETDB1 has been in the spotlight as the key regulator for suppressing innate immunity by limiting the overall abundance of TE transcripts in cancer cells (Guler et al., 2017). Genomic TEs, when derepressed, produce double-stranded RNAs or reverse-transcribed DNAs into the cytoplasm; these atypical nucleic acids should be eliminated by metabolism, and if not removed, they cause an inappropriate activation of nucleic acid-sensing pathways, ultimately inducing the IFNα response and cell death (Zeng et al., 2014) or autoimmunity by their chronic presence (Volkman and Stetson, 2014). The role of nuclear SETDB1 as the primary effector in the repression of TEs, particularly endogenous retrovirus (ERV) and LINE1 copies (Matsui et al., 2010; Liu et al., 2014), can be broadened to its cytoplasmic function for surveillance of these spin-offs of TE transcripts. SETDB1 may indirectly recognize TE-derived nucleic acids and associate with the Argonaute (AGO) protein (Cho et al., 2014) at the start of the RNA*i* process before targeting the genomic TEs of interest in the nucleus to re-repress their transcription and eventually block the IFNα response (Kang, 2018). The benefits of this feedback regulation scenario are that SETDB1 is a well-known TE silencer and, by virtue of its operating mechanism, can efficiently scan the chromosomes for complementary sequences of any transcribed TE-derived sequences. The latter is plausible because SETDB1 can simultaneously access and quickly leaf through a large fraction of the genome through the association of promyelocytic leukemia (PML)-nuclear bodies, as suggested (Cho et al., 2011; Kang, 2015). Consistently, an increase in the number and size of PML-NBs was observed after viral infection and IFN treatment (Regad and Chelbi-Alix, 2001). Additionally, double-stranded RNAs of the TE origin have been demonstrated to induce the production of endogenous small interfering RNAs (siRNAs) and subsequently silence TEs by an RNAi-dependent mechanism in human cells (Yang and Kazazian, 2006). This scenario well harmonizes with our GSEA result, which is as follows: the depletion of genes belonging to the host innate immune response and several autoimmunity-related collections, and the enrichment of genes associated with the RNA*i* and small RNA production in the SETDB1-overrepresented samples (Figures 2, 3). Therefore, results of our meta-analysis of lung SH tumor transcriptomes provided several clues that support the generality of SETDB1 functions for controlling innate immunity and presumably autoimmunity through the surveillance of TE expressions that have been previously proposed in different types of cancer cells or cultured cells.

SETDB1 transcriptionally represses TEs as an effector in the KAP1-built repressive complex (Matsui et al., 2010); therefore, it stands to reason to give the first consideration to these TEs for their expression change in the SH tumor samples. Unfortunately, however, the TE expression data were unavailable in the RNA-seq datasets analyzed in this study; thus, we were unable to investigate the extent to which the genomic TE expression would be affected and altered by SETDB1 overexpression in lung cancers. If the TE data are available, it would be interesting to examine whether the depletion of genes related to innate immunity and autoimmunity-associated collections in the SH samples (Figure 3D) correlates with the transcriptional repression of TEs by SETDB1-involved mechanism and whether the reduced expression of nucleic acid sensors (Figure 3E) may be intertwined in this regulatory circuit.

The lack of DNA methylation data on genomic TEs and other repeated sequences is also a limitation. Compared with the SL samples, the SH samples expressed the three DNA methyltransferases *DNMT1, DNMT3A*, and *DNMT3B* at higher levels, but we were unable to completely understand the impact of DNMT overexpression in lung tumors because the methylome datasets used in this study were derived from the Infinium 450 K array platform, which can identify methylation changes with a single CpG resolution at unique genomic loci (Bibikova et al., 2011) but, in exchange for advantage, may be inadequate in scrutinizing entire genomic repeats and unique sequences in the intergenic regions (Min et al., 2019). It might be important to assess methylation changes over the genomic repeats in lung SH tumors, particularly when the targets of DNMT3A and DNMT3B are known to be pericentric heterochromatin (Okano et al., 1999; Xu et al., 1999; Bachman et al., 2001), intracisternal A particles (IAP) (Walsh et al., 1998; Kaneda et al., 2004; Kato et al., 2007), and many other repeated sequences. Genome-wide DNA methylation studies on lung cancer have mostly focused on the genic promoter regions for cancer-specific biomarkers (Kwon et al., 2012; Bjaanaes et al., 2016). In a previous report (Rauch et al., 2008), the combined bisulfite restriction analysis (COBRA) method was used, in which SCC samples exhibited hypomethylation at genomic repeats, including LINE1s and ERVs. This PCR-based method is still of limited use for studying the entire genome. Therefore, to comprehensively understand the lung cancer methylome and the implication of SETDB1 overexpression, we should first determine the methylation change occurring in the genic loci and in the various genomic repeats that occupy almost half of the genome.

The DMCs found in the ADCs were mostly undermethylated in the SH samples compared with the SL samples, whereas in SCCs, it was the opposite (Figure 6B). The antithetical pattern of change in DNA methylation was interesting because both ADCs and SCCs commonly overrepresented the *DNMT* gene transcripts in the SH samples (Figure 6A). This suggests that although SETDB1 overexpression is related to DNMT overexpression, these proteins may be uncoupled with genomic DNA methylation in the ADCs. The reason for the inverse relationship between DNMT expression and DNA methylation level in the ADCs should be addressed, which, we hope, will be answered when a genome-wide methylation analysis, such as MBD sequencing or whole-genome bisulfite sequencing of lung SH tumor samples, is performed. We hypothesize that abundant transcriptional activators that prevail in the cancer cells preoccupy the must-be-methylated regions in collaboration with other chromatin relaxing epidrivers (such as HATs) and, thus, ward DNMTs off the spots, causing the regions to be passively demethylated. Similarly, the increased expression of *DNMT* genes may be a feedback response to set the dwindled, irrelevant genomic methylation right. In this sense, CGIs, which are frequently hypermethylated in lung (Rauch et al., 2008; Carvalho et al., 2012) and other human cancers, including particularly those associated with tumor suppression and other genome defense pathways (Esteller, 2002), may be important battlegrounds where both transcriptional activators and repressors compete for occupancy. Therefore, it is interesting to notice that the DMCs around the CGIs were undermethylated in the ADC-SH samples, whereas those in the SCC-SH samples were mostly relatively highly methylated, although they appeared to comply with the overall methylation changes of their own types, as seen in the methylation changes in the “Open_sea” regions (Figure 6C).

SETDB1 is increasingly attracting researchers’ attention for brain disorders including Huntington’s disease (Ryu et al., 2006), schizophrenia (Chase et al., 2013), and autism (Cukier et al., 2012), in addition to cancers. If the questions on the action mechanisms and targets of SETDB1 in diseased cells can be solved, it will be possible to build a therapeutic plan for the targets and at multiple levels in the SETDB1 pathogenic pathway.

## Data Availability Statement

Gene expression datasets for TCGA LUAD (https://portal. gdc.cancer.gov/projects/TCGA-LUAD) and lung squamou-cell carcinoma (https://portal.gdc.cancer.gov/projects/TCGA-LUSC) were downloaded from Genomic Data Commons Data Portal (https://portal.gdc.cancer.gov/), and copy number alteration data were obtained from cBioPortal (https://www.cbioportal.org/).

## Author Contributions

Y-KK: conception, design, and administrative support. BM: collection and assembly of data. Y-KK and BM: data analysis and interpretation. Both authors wrote and approved the final manuscript.

## Conflict of Interest

The authors declare that the research was conducted in the absence of any commercial or financial relationships that could be construed as a potential conflict of interest.

## Footnotes

1 https://github.com/jkrijthe/Rtsne

## Abbreviations

ADC: adenocarcinoma
SCC: squamous-cell carcinoma
SCLC: small-cell lung carcinoma
NSCLC: non-small-cell lung carcinoma
SH and SL: SETDB1-high and -low
TCGA: The Cancer Genome Atlas
CNA: copy number alteration
GSEA: gene set enrichment analysis
fGSEA: fast pre-ranked GSEA
DEGs: differentially expressed genes
ES: enrichment score
NES: normalized ES
EMT: epithelial–mesenchymal transition
RNAi: RNA interference
DE: differential enrichment
CpGs: CpG dinucleotides
DMCs: differentially methylated CpGs
CGI: CpG island
ERV: endogenous retrovirus
TE: transposable element.
