# Supplementary material for: SETDB1 Overexpression Sets an Intertumoral Transcriptomic Divergence in Non-small Cell Lung Carcinoma
Source: Front Genet. 2020 Dec 2;11:573515. doi: 10.3389/fgene.2020.573515 (PMC7738479; doi:10.3389/fgene.2020.573515)

Supplementary figure legends

Figure S1. Survival plots for Setdb1-high (red) and -low (black) patients in TCGA ADC (A) and SCC (B). The number of samples used in the analysis was denoted in the plot. P values indicate the statistical significances of log-rank test between survival curves of SETDB1-high and -low groups. Shades around survival curves represent confidence intervals in 95% confidence levels.

Figure S2. Public RNA-seq data (Sato et al.) for lung adenocarcinoma were analyzed based on the *SETDB1* expression levels. Samples were ordered, and 20 of them that exhibited the highest or the lowest SETDB1 expression were extracted to compare their transcriptomes by DESeq2.

A. The SETDB1 expression levels in tumor samples obtained from Sato et al. The box plots show the SETDB1 expression distribution of overall samples (left) and in the top (Top20) or the bottom 20 (Bot20) ranked samples (right). The red dots on the right boxplots represent the mean expression levels. The fold changes between the Top20 and Bot20 samples are denoted under the plot. The PCA plot exhibits differences in the transcriptomes between the Top20 (red) and Bot20 (blue) samples.

B. The number of common DEGs in Sato et al. and this study is depicted as a Venn diagram, and the fold changes and significances for 21 common DEGs are described in the table.

Figure S3. Validation of differential expressions using a quantitative real-time PCR analysis. Differentially expressed genes (DEGs) that were overexpressed in SETDB1-high ADC samples are shown similarly overrepresented in the GFP-SETDB1-expressing A549 cells (SH-A549) compared with the control A549 cells (A549). Target genes were randomly chosen from the DEG list in the Table S2. Fold-change difference of each gene between the SH-ADC and SL-ADC samples is shown for reference. P values are shown (paired-sample t-test in the SH-A549 vs. A549 comparison). Error bars, standard deviation.

Figure S4. Single sample gene enrichment alaysis (GSEA) in SETDB1-high (SH) and SETDB1-low (SL) lung squamous cell carcinoma samples.

Using GSVA, single sample GSEA was performed on three gene collections from MSigDB (v7.0): HALLMARK (A), KEGG (B), and GO:BP (C). Volcano plot shows the distribution and the number of gene sets with differential enrichments (DE; $FDR < 1 \times 10^{-5}$) between the SH and SL samples in each collection; each dot indicates a gene set included in selected MSigDB collections. In the plot, x-axis designates the difference (SH − SL) of enrichment scores between SH and SL and y-axis represents the statistical significance of DE scores; red and blue dots indicate gene sets that are enriched and depleted in SH samples, respectively. Heatmaps show the differential enrichment among individual SH and SL samples. Samples are hierarchically clustered on X-axis (SH, red; SL, blue), and significant DE gene sets are shown on y-axis. Black bars on the left represent the gene sets mentioned in Figure 3, and the gene set names are denoted on the right. Colors in GSVA score bar indicate enrichment scores in individual samples.

Figure S5. The quantification of the differences in the gene expression levels between SETDB1-high and SETDB1-low tumors of adenocarcinoma (ADC) and squamous-cell carcinoma (SCC) by calculating the root-mean-square deviation (RMSD) of tumor samples of each cancer type from normal samples. The RMSD values are indicated on the bars. The deviation in expression level is the highest in DNA methylation (DNAm) category genes, followed by histone lysine methylation (K meth) category genes. K Meth and K demeth, histone lysine methylation and demethylation; R meth and histone arginine methylation; Acetyl and Deacetyl, histone acetylation, and deacetylation; and Ubiquit and histone ubiquitination.

Supplementary Figure S1

ADC

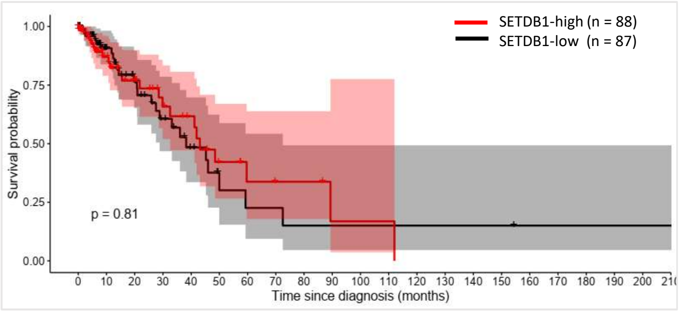

SCC

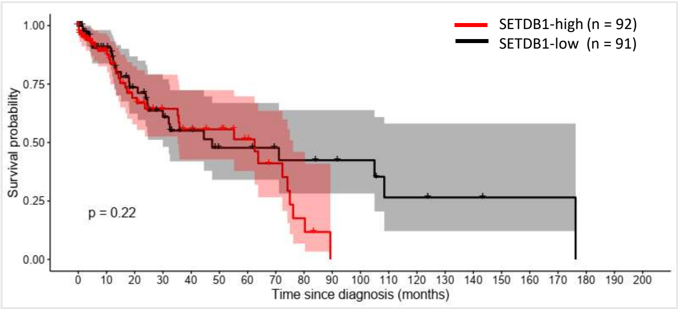

Supplementary Figure S2

A

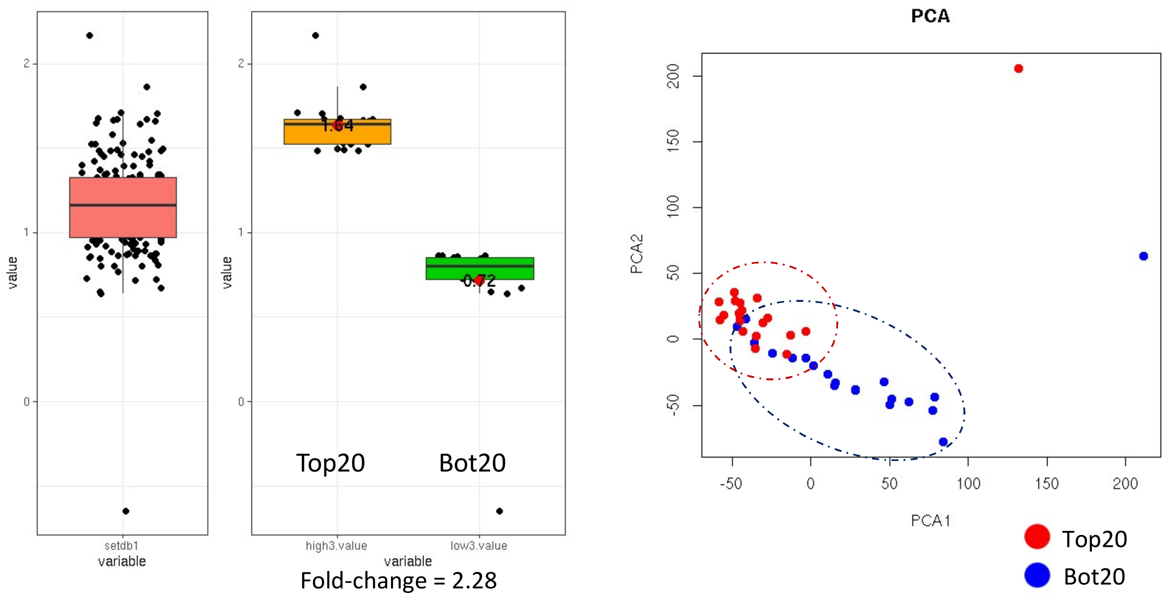

B

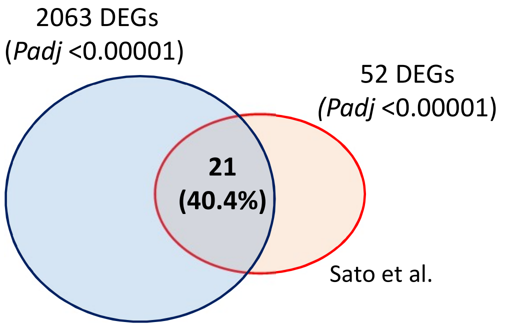

Symbol	This study			Sato et al.		
	log2FC	*P* value	*Padj*	log2FC	p value	Padj
SETDB1	1.432	4.6E-246	1.9E-241	0.921	2.9E-14	5.7E-10
INTS3	1.036	1.9E-49	1.1E-46	0.665	1.5E-13	1.4E-09
PI4KB	0.789	1.3E-56	1.2E-53	0.551	1.8E-11	1.2E-07
BCL9	1.259	1.5E-54	1.2E-51	0.694	4.2E-11	2.1E-07
CLUH	0.634	3.1E-12	5.3E-11	0.630	7.2E-11	2.8E-07
CGN	1.192	3.0E-16	9.6E-15	0.934	5.5E-10	9.9E-07
ANO8	0.647	2.2E-08	1.9E-07	0.730	1.2E-09	1.6E-06
HCN3	1.178	2.1E-22	1.5E-20	1.035	1.9E-09	2.2E-06
PELP1	0.627	1.1E-15	3.2E-14	0.601	2.3E-09	2.3E-06
RRNAD1	0.655	1.8E-19	9.2E-18	0.640	2.7E-09	2.3E-06
RBX1	-0.690	1.3E-14	3.2E-13	-0.428	3.6E-09	2.8E-06
PPOX	0.617	4.3E-12	7.0E-11	0.580	3.8E-09	2.9E-06
CDK5RAP3	0.512	6.6E-09	6.1E-08	0.712	3.9E-09	2.9E-06
UPF2	0.584	1.1E-17	4.4E-16	0.373	6.2E-09	3.8E-06
TOP3B	0.773	7.9E-14	1.7E-12	0.584	6.9E-09	4.1E-06
RNF31	0.375	5.1E-08	4.0E-07	0.521	8.7E-09	4.6E-06
SUPV3L1	0.375	1.9E-11	2.8E-10	0.461	9.5E-09	4.9E-06
ZC3H3	0.480	3.8E-10	4.5E-09	0.574	1.4E-08	6.1E-06
ARHGAP39	0.723	3.5E-09	3.4E-08	0.946	1.5E-08	6.1E-06
SETD1A	0.424	1.1E-08	9.5E-08	0.512	1.4E-08	6.1E-06
KAT2A	0.618	1.4E-12	2.6E-11	0.723	1.7E-08	6.7E-06

Supplementary Figure S3

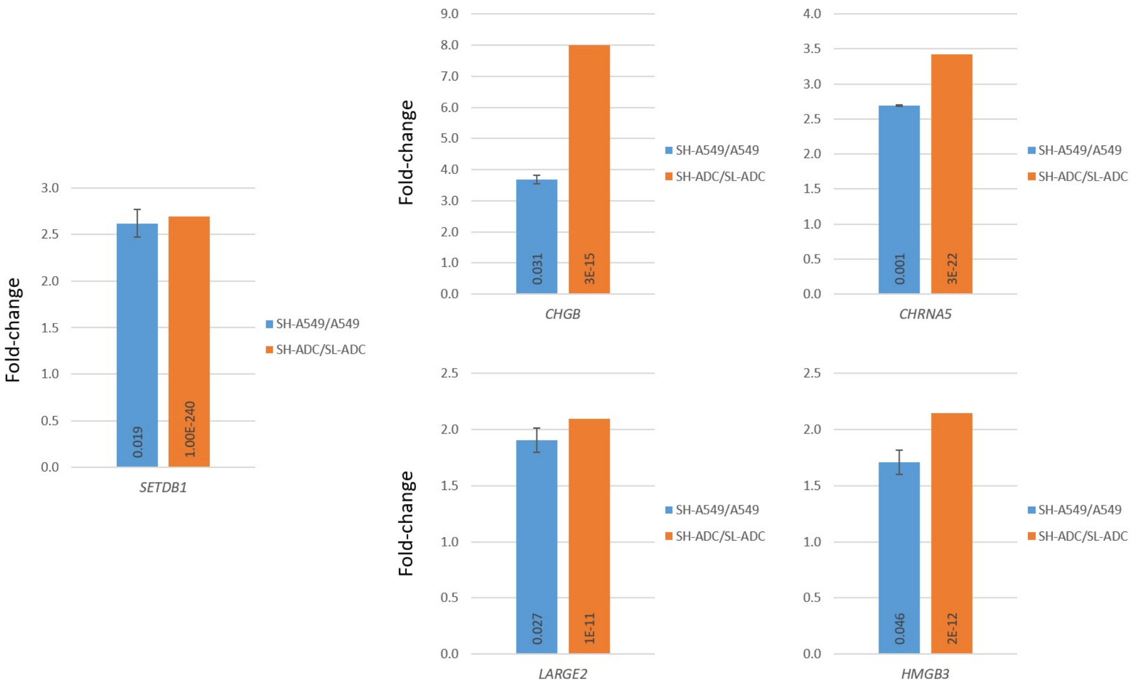

Supplementary Figure S4

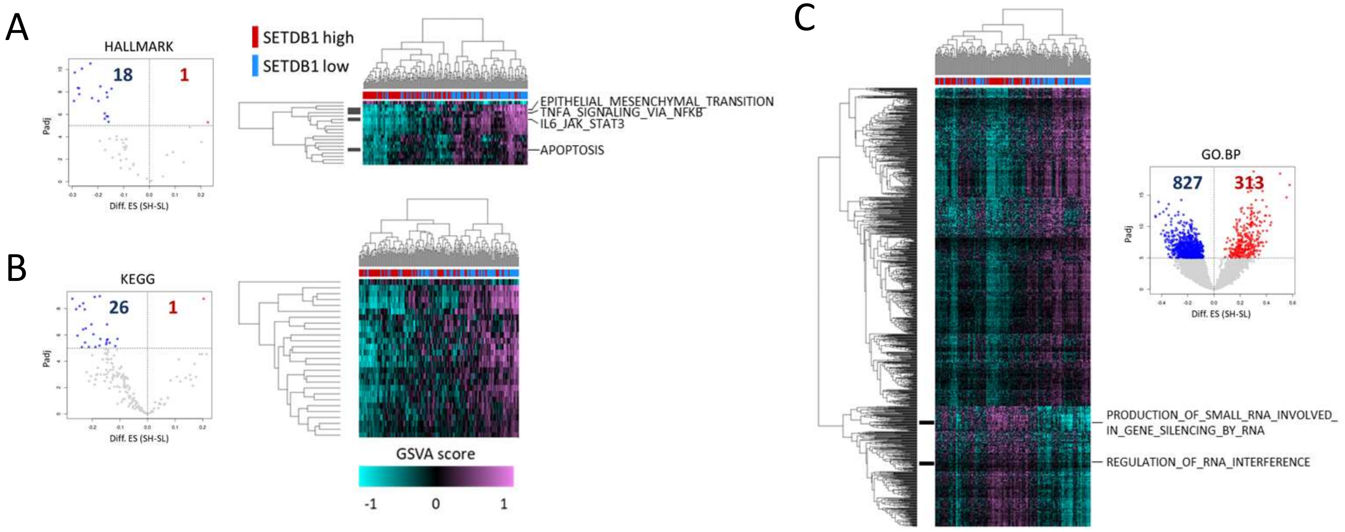

Supplementary Figure S5

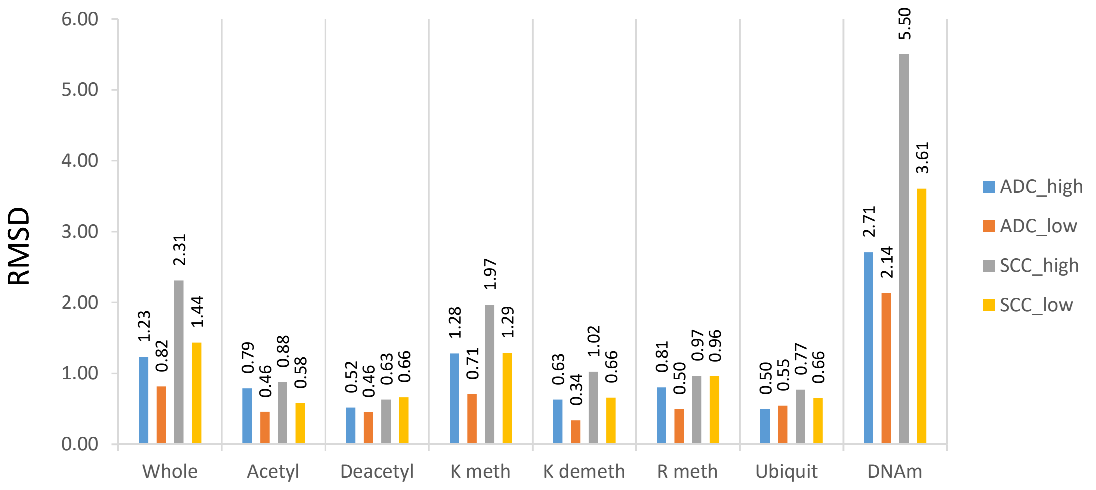

Supplement: Supplementary file 1 [file Presentation_1.pdf]
